# High-Temperature Flow Behavior and Energy Consumption of Supercritical CO_2_ Sealing Film Influenced by Different Surface Grooves

**DOI:** 10.3390/ma16227129

**Published:** 2023-11-11

**Authors:** Jing Yang, Shuaiyu Wang, Shaoxian Bai

**Affiliations:** College of Mechanical Engineering, Zhejiang University of Technology, Hangzhou 310032, China; wsy980713@163.com

**Keywords:** supercritical CO_2_, flow behavior, energy consumption, face seals, surface grooves

## Abstract

The Brayton cycle system, as a closed cycle working under high-temperature, high-pressure and high-speed conditions, presents significant prospects in many fields. However, the flow behavior and energy efficiency of supercritical CO_2_ is severely influenced by the structures of face seals and the sealing temperature, especially when the sealing gas experiment is the supercritical transformation process. Therefore, a numerical model was established to investigate the high-temperature flow behavior and energy consumption of face seals with different surface grooves. The effects of the operation parameters and groove structure on the temperature distribution and sealing performance are further studied. The obtained results show that the supercritical effect of the gas film has a more obvious influence on the flow velocity *u*_θ_ than *u*_r_. Moreover, it can be found that the temperature distribution, heat dissipation and leakage rate of the gas face seals present a dramatic change when the working condition exceeds the supercritical point. For the spiral groove, the change rate of heat dissipation becomes larger, from 3.6% to 8.1%, with the increase in sealing pressure from 15 to 50 MPa, when the temperature grows from 300 to 320 K. Meanwhile, the open force maintains a stable state with the increasing temperature and pressure even at the supercritical point. The proposed model could provide a theoretical basis for seal design with different grooves on the supercritical change range in the future.

## 1. Introduction

In recent years, the supercritical carbon dioxide (sCO_2_) Brayton cycle has attracted much attention in practical engineering fields due to its higher efficiency [1,2]. Many works have proven that the gas properties of CO_2_ present an obvious change when they experience the supercritical transition, which may ultimately affect the heat transfer properties and flow behaviors of CO_2_ [3,4,5,6,7]. As the key structure of the sCO_2_ system, gas face seals and their performance [8], structure [9], optimization [10] and design [11] have been investigated by many researchers. In these studies, the pressure and temperature conditions are relatively low compared with the rapid development of high-parameter mechanical equipment. Therefore, this study aims to investigate the flow behavior and thermal properties of sCO_2_ under high-pressure and -temperature conditions, which might provide guidance for the design of gas face seals under harsh conditions in the future. 

Early in 1960, Schmidit [12] found that a higher heat transfer coefficient would be observed when the working condition was near the critical point of supercritical fluid. Due to this advantage, sCO_2_ has been used in many fields, including as a compressor, turbine and seal [13,14,15,16,17,18,19]. For example, Pei et al. [20] proposed a numerical model to study the flow behavior and performance of a centrifugal compressor influenced by leading edge profiles. It was found that the suction peak at the leading edge was reduced with the growth of the long-short ration of the leading edge. Du et al. [3] focused on the axial-flow turbine in sCO_2_ power cycle and tip leakage. They found that, compared with Labyrinth, the leakage of dry gas seal was reduced by 99.38%, and the overall performance improved substantially. As the supercritical point of CO_2_ (*T*_c_ = 305.128 °C, *P*_c_ = 7.3773 MPa) is easier to reach, some researchers have considered it to be safe to replace other gases to obtain the physical properties under the supercritical state using an experimental method [21]. 

Groove treatment on the rotor surface is a widely used method to improve the heat transfer characteristics and to reduce energy consumption for gas face seals [22,23]. To date, many works have focused on the hydrodynamic effect and sealing performance of gas face seals as influenced by the groove type [24,25,26,27,28,29]. For example, Zhu and Bai [9] investigated the thermoelastohydrodynamic characteristics and sealing performance of sCO_2_ face seals with a T groove. They found that the face distortion induced by increasing the pressure may lead to divergent clearance and reduce the opening force when the temperature was close to the critical transition point, while the distortions generated by increasing the temperature may lead to convergent clearance when the gas is in the supercritical state. Ding and Lu [30] used the theoretical method and experiment to investigate the sealing film temperature of gas face seals with a spiral groove under high-speed and -pressure conditions. According to the temperature distribution of the gas film, they found that the temperature at the root radius is larger than that at the inner radius and outer radius. The increase in temperature from the inner side to the root radius could be attributed to the thermal dissipation induced by the pressure drop of the surface groove. 

However, the thermal properties and sealing performance of gas face seals can be influenced not only when the gas state closes the supercritical point, but also when it exceeds this point. Therefore, this paper aims to analyze the flow behavior and energy consumption of gas face seals with different surface grooves under high-temperature and -pressure conditions. First, the influences of the supercritical effect and surface grooves on the flow velocity, temperature distribution and heat dissipation are investigated. Then, the sealing performance is studied with an increase in the sealing pressure and temperature. Finally, the conclusions will be summarized. 

## 2. Numerical Model 

**Figure 1:**
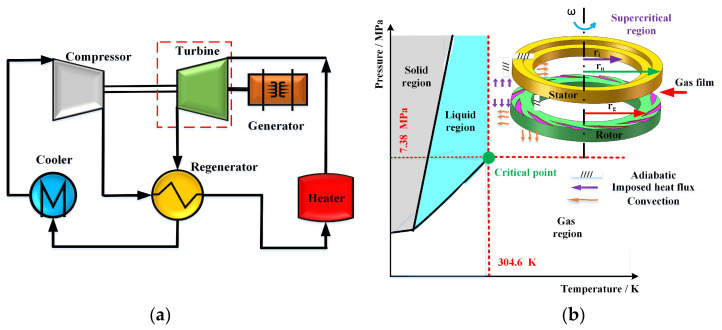
The schematic diagram and boundary condition of gas face seals in Brayton cycle. (**a**) Brayton cycle of supercritical CO_2._ (**b**) Gas face seal with spiral groove and boundary condition.

Gas face seals are sealing structures of turbine commonly used in the Brayton cycle due to their low wear and high stability, as shown in Figure 1. As the physical parameters of CO_2_ presents an obvious change when the gas state transforms from the standard to the supercritical state, the flow behavior of this gas film may have a significant influence on the sealing structure. A numerical model is needed to grasp the flow behavior and energy consumption of gas face seals. The detailed model development is introduced as follows. 

### 2.1. Governing Equations

In order to analyze the flow behavior and energy consumption of gas face seals when the sealing gas experiences the transition from the stead state to the supercritical state with an increase in the sealing pressure and temperature, the governing equation needs to take the real gas parameters, solid heat conduction and energy transform into consideration based on the Reynolds equation. As the mass of gas is small, the centrifugal effect is very weak and can be ignored in this numerical model. The detailed Reynolds equation of the polar coordinate system is as follows [31]:

(1) $$\frac{\partial }{r\partial \theta }\left(\frac{h^{3}\rho }{\eta }\frac{\partial p}{r\partial \theta }\right)+\frac{\partial }{r\partial r}\left(\frac{h^{3}r\rho }{\eta }\frac{\partial p}{\partial r}\right)=6\omega \frac{\partial (\rho h)}{\partial \theta }+12\frac{\partial (\rho h)}{\partial t}$$

where *η* and *ρ* are the viscosity and the density of the sealing gas, respectively; *p* and *h* are the pressure and the thickness of the sealing gas; *r* and *θ* are the polar coordinates. 

The gas pressure *p* can be obtained by:

*p* = *εc_p_ρi_d_E_m_* (2)

where *ε* is the compressibility coefficient of the sealing gas, *i_d_* is the freedom degree of the gas molecular and *c*_p_ can be calculated by 2/*i_d_*; *E_m_* is the energy per degree of freedom for the gas molecular.

For the gas face seals, the viscosity-pressure and temperature-viscosity effect of the sealing gas need to be considered in the energy equation. Moreover, the surface elastic deformation and thermal deformation are also very important for the accuracy of the numerical calculation. Therefore, the energy equation of the sealing film can be written [31]:

(3) $$\begin{array}{l} \left(-\frac{h^{3}}{12\eta }\frac{\partial p}{r\partial \theta }+\frac{\omega rh}{2}\right)\frac{\partial T}{r\partial \theta }-\frac{h^{3}}{12\eta }\frac{\partial p}{\partial r}\frac{\partial T}{\partial r}\\ =\frac{\eta \omega^{2}r^{2}}{h\rho c_{v}}-\frac{h^{3}}{12\eta \rho c_{v}}\left[{\left(\frac{\partial p}{r\partial \theta }\right)}^{2}+{\left(\frac{\partial p}{\partial r}\right)}^{2}\right]+\frac{k_{\mathrm{gs}1}}{\rho c_{v}}(T_{s1}-T)+\frac{k_{\mathrm{gs}2}}{\rho c_{v}}(T_{s2}-T) \end{array}$$

where *ω* is the rotational speed of the rotor, *T* is the temperature of the sealing film, *c*_v_ is the specific heat capacity, *k*_gs1_ and *k*_gs2_ are the convection heat transfer coefficients, *T*_s1_ and *T*_s2_ are the solid surface temperatures in the lubrication regime. Here, the rotor and stator are made of stainless steel and carbon. The detailed material parameters are listed in Table 1.

Taking the thermal deformation of the end face and the surface groove depth into consideration, the governing equation of the gas thickness can be obtained by:

(4) $$h\left(r,\theta \right)=h_{\min}+h_{p}\left(r,\theta \right)+h_{\mathrm{Deform}}\left(r,\theta \right)$$

where *h*_min_ is the minimum sealing clearance, *h*_p_ is the groove depth and *h*_deform_ is the deformation induced by the thermal effect. 

Considering the heat transfer behavior between the stator and rotor, the heat conduction equation at the polar coordinate system can be expressed as:

(5) $$\begin{array}{cc} \frac{\partial^{2}T_{s}}{r^{2}\partial \theta^{2}}+\frac{\partial }{r\partial r}\left(r\frac{\partial T_{s}}{\partial r}\right)+\frac{\partial^{2}T_{s}}{\partial z^{2}}=0 & \;\;\;\;\;\;\;\;\;\;\;\;\;\;\;\;\;\;\;\;\;\;\;\;\;\;\;\;\;\;\;\;\;\;\;\;\;\;\;\;\;\;\;\;\;\;\;\;\mathrm{for}\;\mathrm{stator} \end{array}$$

(6) $$\frac{k_{c2}}{\rho_{s2}c_{s2}}\left[\frac{\partial^{2}T_{s}}{r^{2}\partial \theta^{2}}+\frac{1}{r}\frac{\partial }{\partial r}\left(r\frac{\partial T_{s}}{\partial r}\right)+\frac{\partial^{2}T_{s}}{\partial z^{2}}\right]=\omega \frac{\partial T_{s}}{\partial t}\;\;\;\;\;\;\;\;\;\;\;\;\;\;\;\;\;\mathrm{for}\;\mathrm{rotor}$$

where *T*_s_ is the temperature of the solid; *k*_c2,_ *ρ_s_*_2_ and *c*_s2_ are the heat transfer coefficients of the friction pair, density and specific heat capacity, respectively.

The thermal boundary between the sealing film and sealing rings was assumed as imposed heat flux, convection and adiabatic, shown in Figure 1b. It needs to satisfy the following interface equations:

(7) $$-k_{c1}{\left(\frac{\partial T_{s}}{\partial n}\right)}_{s}=k_{s1}\left(T_{s1}-T\right)$$

(8) $$-k_{c2}{\left(\frac{\partial T_{s}}{\partial n}\right)}_{s}=k_{s2}\left(T_{s2}-T\right)$$

where *k*_s_ is the convective heat transfer coefficient. 

### 2.2. Boundary Conditions

As the surface grooves are distributed along the axis and radial directions averagely, the simulated region can select a periodic area in order to reduce the simulating time. Based on this condition, the periodic pressure boundary condition and periodic temperature boundary condition can be described, respectively, as follows:

(9) $$p\left(r,\theta =\pi /N\right)=p\left(r,\theta =-\pi /N\right)$$

(10) $$T\left(r,\theta =\pi /N\right)=T\left(r,\theta =-\pi /N\right)$$

where *N* is the groove number of surface grooves along the radial direction. 

### 2.3. Analysis Parameters

The flow behavior and thermal properties of the sealing film can influence the sealing performance of gas face seals. Based on the practical application of gas face seals, the working parameters selected in this study are listed in Table 2. When the sealing pressure and film thickness are calculated, the flow velocities *u_θ_* and *u_r_* are obtained as follows:

(11) $$u\theta =\frac{1}{2\eta }\frac{\partial p}{r\partial \theta }(z^{2}-zh)+\frac{\omega rz}{h}$$

(12) $$ur=\frac{1}{2\eta }\frac{\partial p}{\partial r}(z^{2}-zh)$$

The friction torque *M* of the gas film are generally used to analyze the heat transfer behavior of gas face seals. The detailed expression of *M* can be written by:

(13) $$M_{0}=\iint \tau \left|{}_{z=0}r^{2}\right.drd\theta$$

(14) $$M_{h}=\iint \tau \left|{}_{z=h}r^{2}\right.drd\theta$$

where the shear stress of the lubricating film can be calculated by $\tau =\frac{1}{2}\frac{\partial p}{r\partial \theta }(2z-h)+\frac{\omega r\eta }{h}$.

Then, the heat dissipation can be calculated by the following expression:

(15) $$Q_{d}=\iint \omega Mdrd\theta$$

Commonly, the sealing performance parameters are the leakage rate *Q* and opening force *F*, which can be defined, respectively, as follows:

(16) $$Q=\frac{h^{3}r\rho }{12\eta }\int_{0}^{2\pi }\frac{\partial p}{\partial r}d\theta$$

(17) $$F=\int_{0}^{2\pi }\int_{r_{i}}^{r_{o}}prdrd\theta$$

### 2.4. Structure of Surface Groove

**Figure 2:**
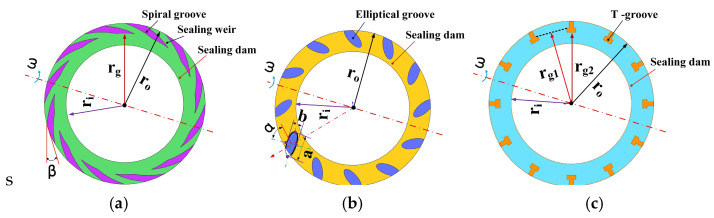
The structure parameters of three surface groove. (**a**) spiral groove. (**b**) elliptical groove. (**c**) T groove.

At present, surface groove technology is widely used in gas face seals. Many studies have been conducted to prove that grooves can significantly improve the hydrodynamic effect and sealing performance. According to different working conditions, the suitable surface groove may be designed to satisfy the engineering requirement. In this study, the commonly used groove structures, including the spiral groove, elliptical groove and T groove, are selected to investigate the flow behavior and energy consumption of gas face seals, shown in Figure 2. In order to compare the simulation results for these three kinds of grooves, the outer radius *r*_0_, inter radius *r*_i_ and root radius *r*_g_ of these grooves are set as 70 mm, 50 mm and 55 mm, respectively. The number of grooves is fixed at 12. The spiral angle of the spiral groove is 18˚ and the slope angle of the elliptical groove is 45°. For different surface grooves, the root radius *r*_g_ of the sealing ring is the most important factor to determine the sealing performance; then, the same root radius of these three kinds of surface groove was used in this simulation.

### 2.5. Numerical Method

In the proposed model, the finite difference method is applied to obtain the film pressure, film temperature and ring temperature. By fitting a discrete difference scheme of governing equations, the flow conservation was guaranteed. The mesh density of the gas film was set as 60 × 60 × 25, which is enough to ensure the accuracy of the simulation based on the previous work. 

**Figure 3:**
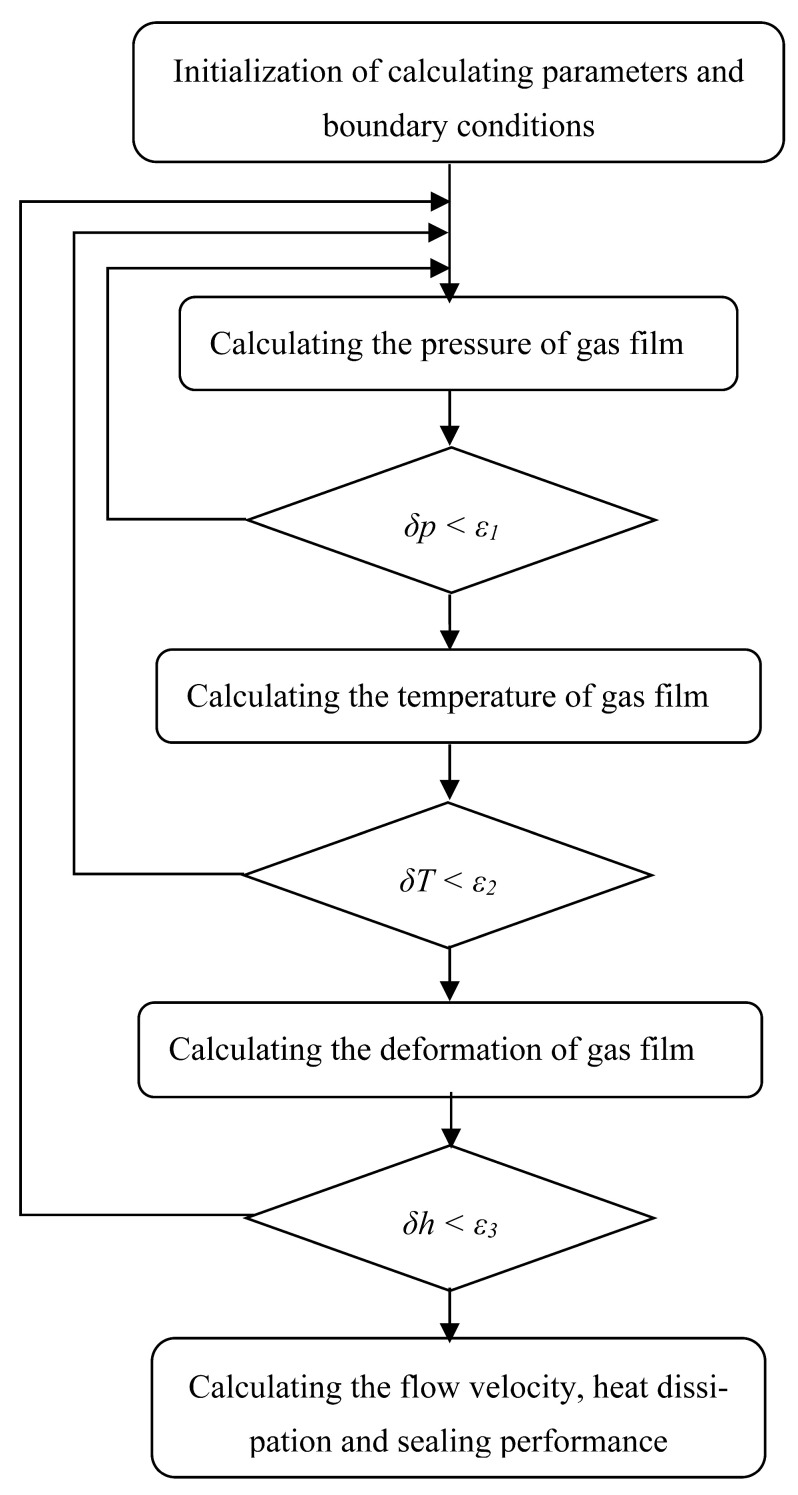
The numerical procedure of proposed model.

When the structure parameters, materials parameters and working conditions of the gas face seals with different surface groove are initiated, the pressure distribution of the gas film will be calculated based on the Reynolds equation. When the gas pressure convergences, the temperature distribution of the gas film is calculated based on the energy equation. If the pressure and temperature change between two iteration processes is less than the convergence criterion, the iteration process will end, and the pressure and temperature will be obtained. Then, according to the formulae, the heat dissipation, flow velocities, open force and leakage rate will be calculated. The detailed procedure of this calculating model is presented in Figure 3. 

### 2.6. Model Validation

**Figure 4:**
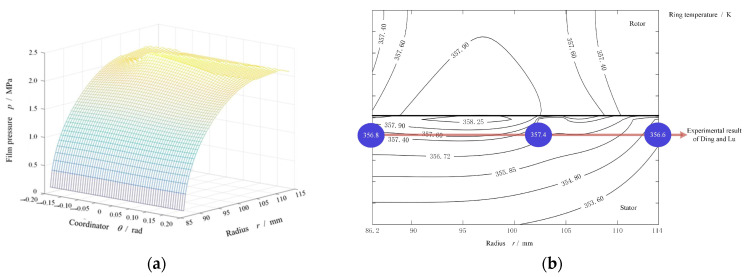
Pressure distribution of face seals with spiral grooves and the compared results between proposed model and Ding and Lu’s experimental work. (**a**) pressure distribution. (**b**) temperature distribution.

In order to validate the proposed model, the temperature distribution of Ding and Lu [30] was used as a comparison with the temperature contour of the proposed model, based on the same parameters listed in Table 3. The pressure distribution under these parameters was calculated, and is plotted in Figure 4a. It was found that along the increasing direction of the radius from 86.2 to 114 mm, the pressure distribution increased and the value at the outer radius exceeded the sealing pressure due to the existence of the grooves. Based on the pressure distribution, the temperature distribution of the stator and rotor was calculated, as shown in Figure 4b. At the same time, the discrete temperature values of the experimental work [30] were added into this figure using bull ball. It can be found that these discrete temperature values were in good agreement with the simulated temperature contour of the proposed model.

## 3. Result Discussion of Flow Behavior

As is well known, the viscosity, density and heat capacity of gas will change when the gas pressure and temperature are close to the supercritical point, which is bound to affect the sealing performance of the gas face seals eventually. In this section, the flow behavior of gas face seals with different surface grooves is investigated when the working conditions increase to the supercritical point and then exceed this point. 

**Figure 5:**
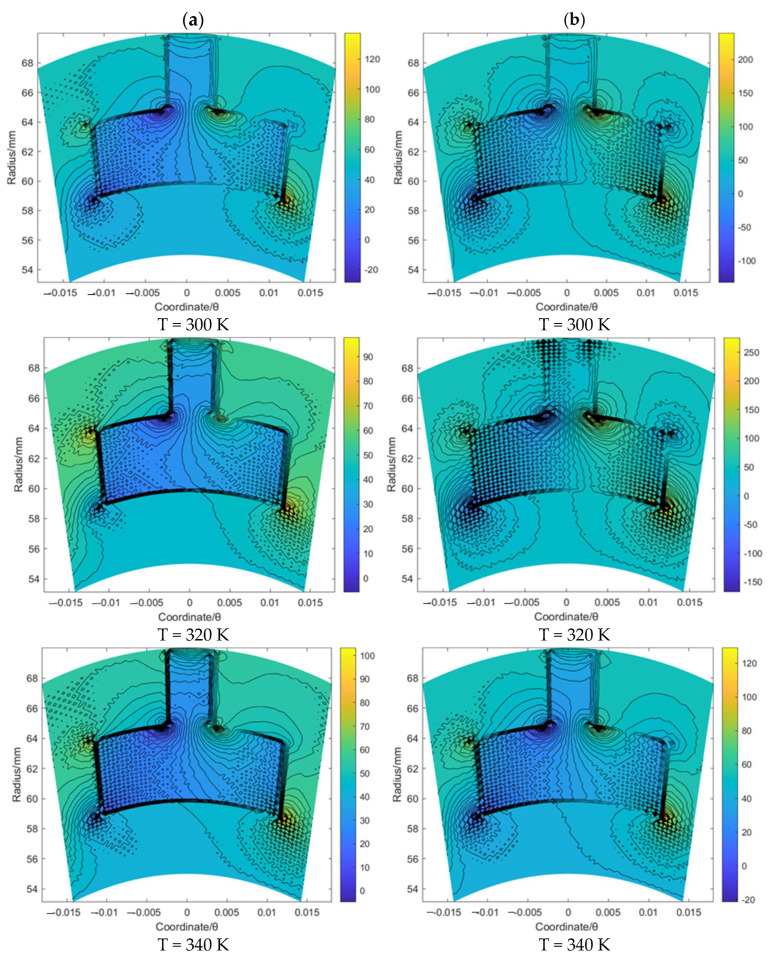
The flow velocity *u_θ_* contour (m/s) of gas face seals with T groove with the increase in temperature for cases: (**a**) *p*_0_ = 20 MPa and (**b**) *p*_0_ = 50 MPa.

**Figure 6:**
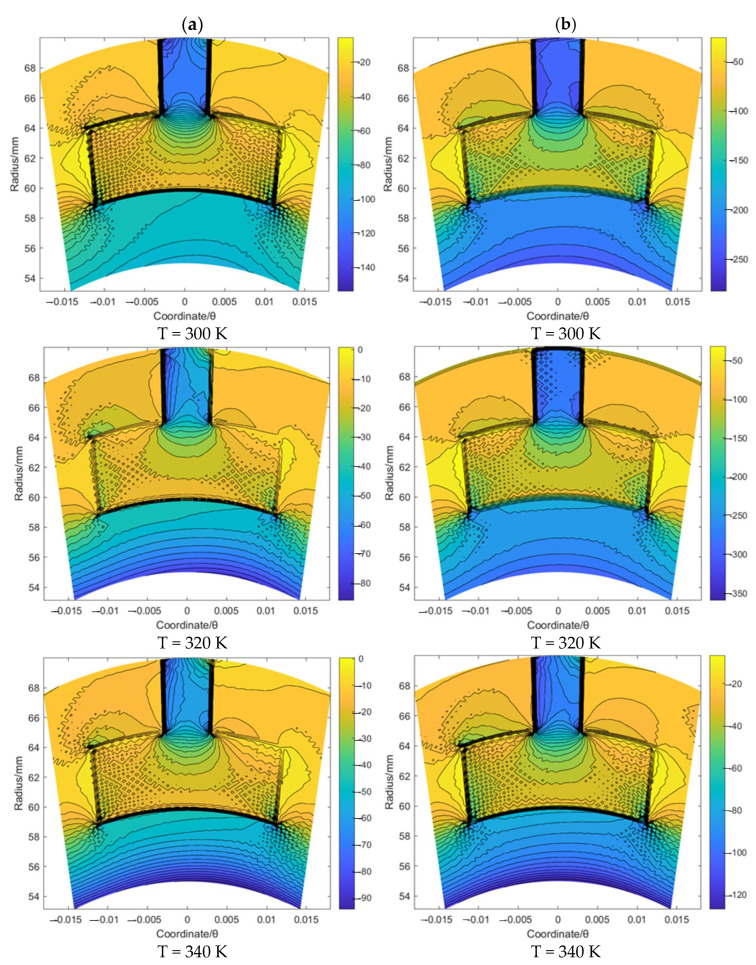
The flow velocity *u_r_* contour (m/s) of gas face seals with T groove with the increase in temperature for cases: (**a**) *p*_0_ = 20 MPa and (**b**) *p*_0_ = 50 MPa.

Figure 5 and Figure 6 illustrate the flow velocity *u_θ_* and *u_r_* of the gas film for the T groove with an increase in the sealing temperature from 300 K to 340 K when the sealing pressure is set as 20 MPa and 50 MPa, respectively. It was found that the boundary of the T groove has an obvious hindrance to the gas flow. Moreover, according to Figure 5, the maximum value of the flow velocity *u_θ_* with the growth in the sealing pressure from 20 to 50 MPa was increased from about 118.5 to 245.6 m/s, 97.5 to 271.2 m/s and 103.1 to 129.5 m/s for the cases of T = 300 K, 320 K and 340 K, respectively. The change rate for T = 320 K is more obvious than that for the other two cases. Based on the simulation result of the flow velocity *u_r_*, as shown in Figure 6, the maximum value of the flow velocity *u_r_* with the growth in the sealing pressure from 20 to 50 MPa was increased from about −5.5 to −24.6 m/s, 0.9 to 31.2 m/s and 0.1 to −5.5 m/s for the cases of T = 300 K, 320 K and 340 K, respectively. The negative value of the flow velocity *u_r_* denotes the gas flow from the outer radius to the inner radius. These results suggest that the supercritical effect of CO_2_ might have a significant influence on the flow behavior of the sealing film, especially when the temperature exceeds the supercritical point. In addition, it was also found that the change range of *u_θ_* and *u_r_* for T = 320 K was much wider than that for T = 300 K and T = 340 K under the same working temperature, which may have also been induced by the supercritical effect.

**Figure 7:**
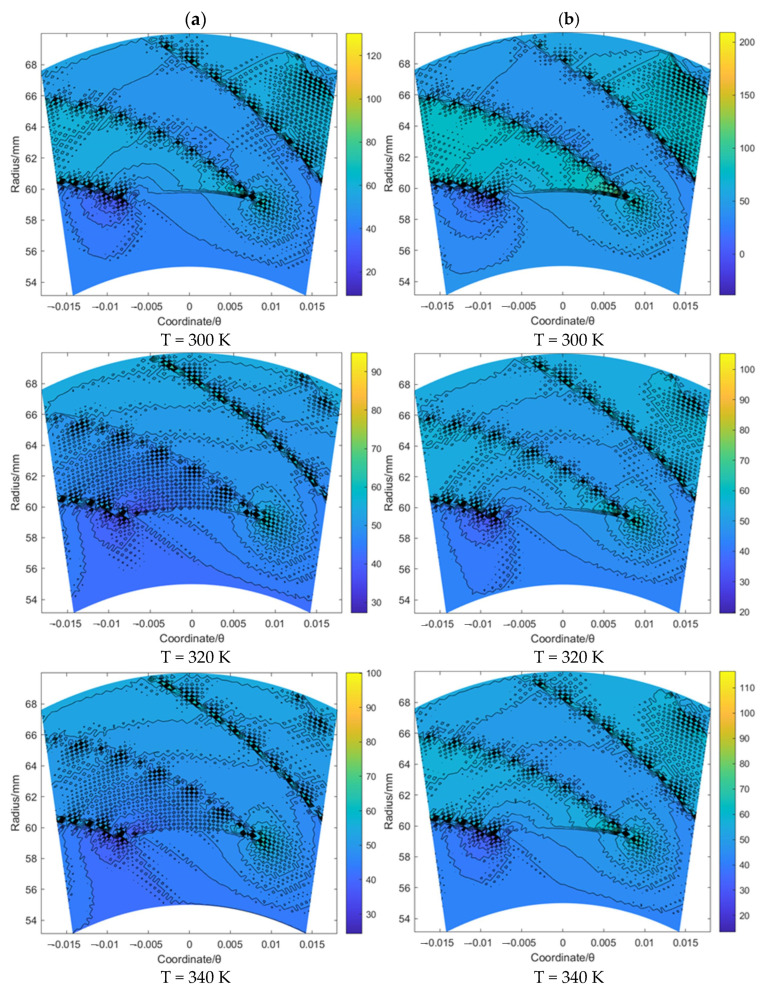
The flow velocity *u_θ_* contour (m/s) of gas face seals with spiral groove with the increase in temperature for cases: (**a**) *p*_0_ = 20 MPa and (**b**) *p*_0_ = 50 MPa.

**Figure 8:**
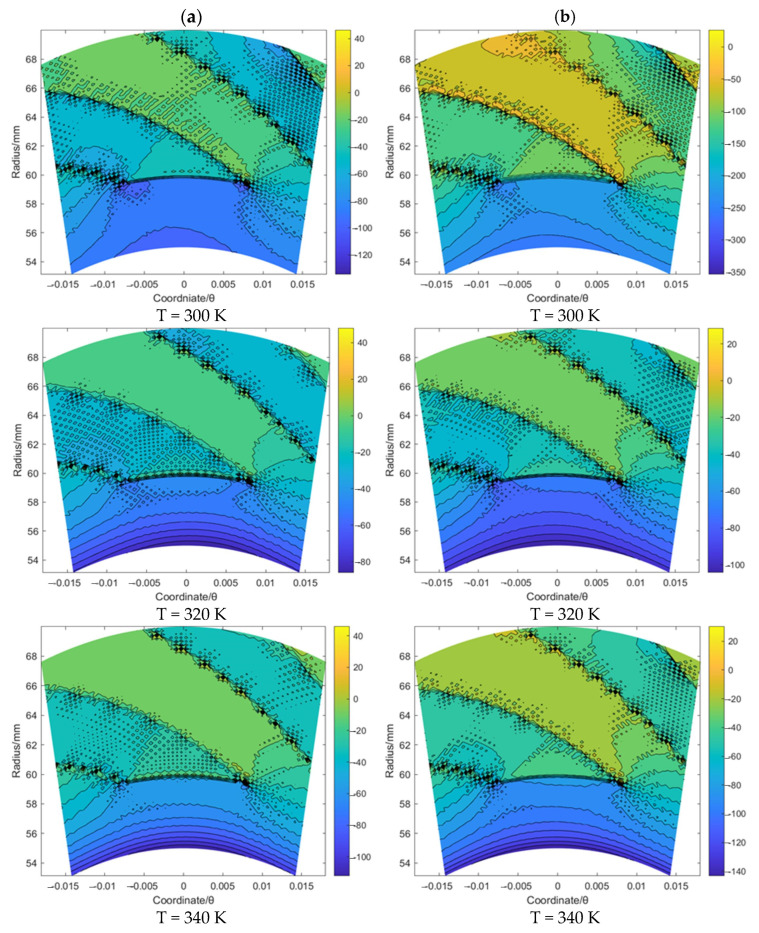
The flow velocity *u_r_* contour (m/s) of gas face seals with spiral groove with the increase in temperature for cases: (**a**) *p*_0_ = 20 MPa and (**b**) *p*_0_ = 50 MPa.

**Figure 9:**
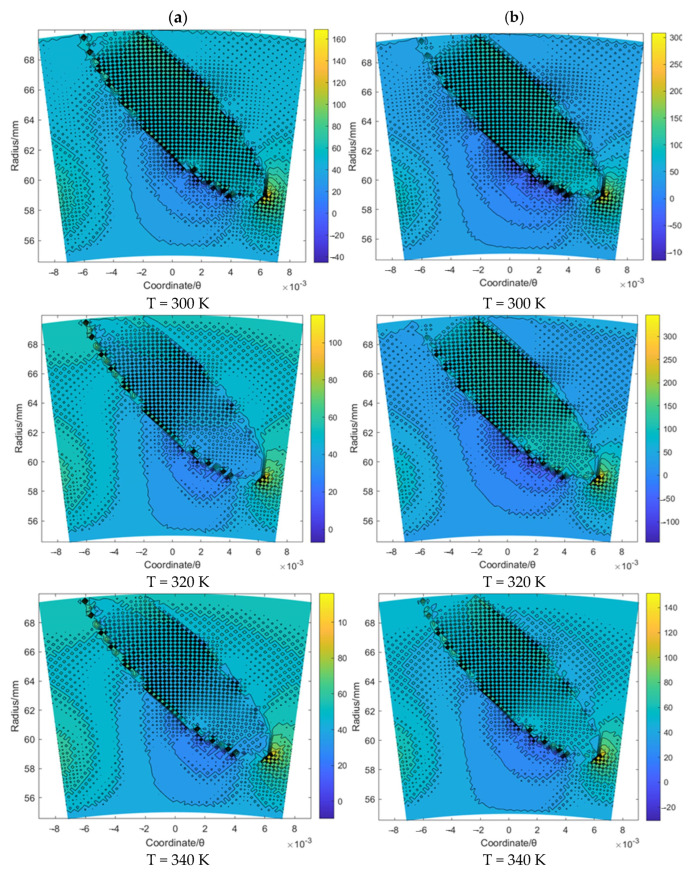
The flow velocity *u_θ_* contour (m/s) of gas face seals with elliptical groove with the increase in temperature for cases: (**a**) *p*_0_ = 20 MPa and (**b**) *p*_0_ = 50 MPa.

**Figure 10:**
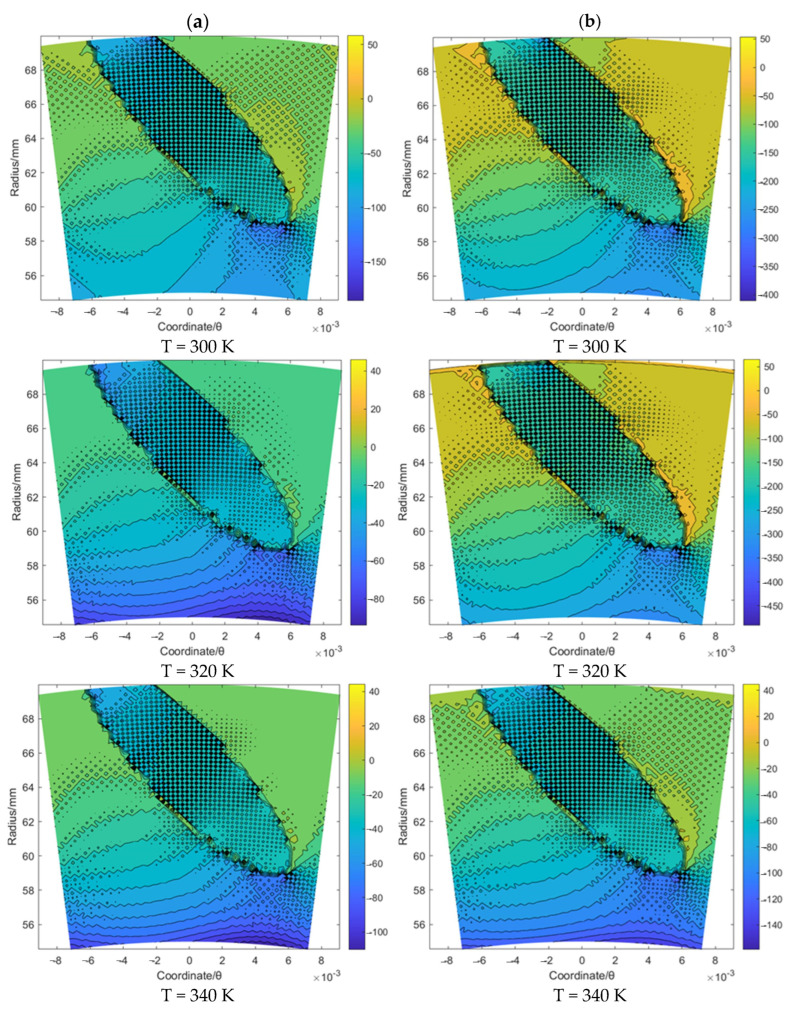
The flow velocity *u_r_* contour (m/s) of gas face seals with elliptical groove with the increase in temperature for cases: (**a**) *p*_0_ = 20 MPa and (**b**) *p*_0_ = 50 MPa.

Based on the same procedure, the flow velocity *u_θ_* and *u_r_* of the gas film for the spiral groove and elliptical groove with an increase in the sealing temperature from 300 K to 340 K when the sealing pressure is set as 20 MPa and 50 MPa, respectively, was studied. According to Figure 7 and Figure 8, for the spiral groove, the obtained results shows that the change range of the flow velocity *u_θ_* and *u_r_* for the T = 300 K case is larger than that for the other two cases. This phenomenon may prove that the flow velocity change influenced by the supercritical effect is stronger before the temperature reaches the supercritical point than when it exceeds this point. According to Figure 9 and Figure 10, for the elliptical groove, the change rule of the flow velocity for the gas face seals with an elliptical groove is more similar to that with the T groove. To be specific, the supercritical effect of gas on the flow velocity for the T groove is more obvious than that for the elliptical groove. 

According to the velocity *u_r_* and *u_θ_* contour of the gas face seals with the T groove, spiral groove and elliptical groove, the following main conclusions of the flow behavior for these three kinds of grooves can be made: (1) the velocity *u_θ_* and *u_r_* will be impeded by the groove edge; (2) the velocity *u_θ_* along the circumference direction remains almost stable; (3) the velocity *u_r_* shows an obvious reverse flow with the growth of the sealing pressure, and this effect is stronger for the elliptical groove than that for the T groove and spiral groove.

## 4. Result Discussion of Temperature Distribution

**Figure 11:**
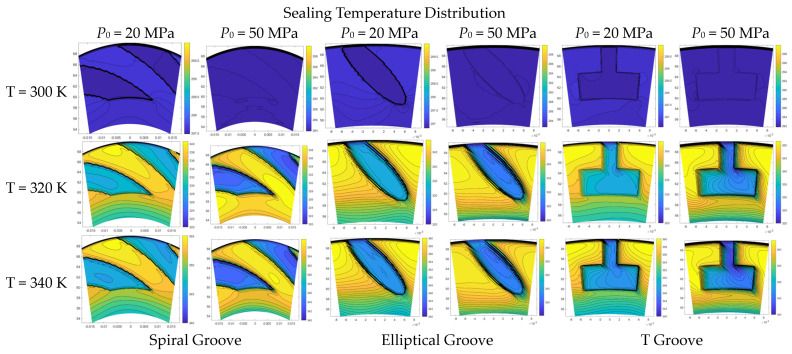
The temperature contours of sealing film with spiral groove, elliptical groove and T groove under different working conditions.

The flow properties of the sealing film have a direct influence on its temperature distribution as the heat can transform with the flow behavior. Therefore, this section mainly investigates the temperature distribution of the sealing film as influenced by the supercritical effect and surface grooves. The simulated results of the temperature distribution are shown in Figure 11; it can be seen that the maximum temperature of the sealing film is slightly smaller than the working condition with the increase in the sealing pressure from 20 to 50 MPa for the three kinds of surface grooves under the sealing temperature T = 300 K. With the growth in temperature, the gas satisfies the supercritical condition. The maximum temperature exceeds the corresponding sealing temperature. For example, when T = 340 K, the maximum temperature of the sealing film increases from 355.05 K to 356.95 K with the growth in pressure from 20 to 50 MPa. Moreover, when the temperature and pressure are the same, the temperature rise of the sealing film with the elliptical groove and T groove is obviously higher than that with the spiral groove.

Moreover, it can be found that the temperature change along the circumference direction is slight, with the exception of the groove area. While the temperature of the sealing film gradually increases along the radius direction, the temperature at the groove area is smaller than that at the non-groove area. At the edge of the groove, an obvious reduction in the temperature is seen from the non-groove area to the groove area.

## 5. Result Discussion of Energy Consumption 

When the gas flows from the outer side to inner side along the radial direction, this film may produce friction heat induced by the motion between the film and ring surface. The energy consumption by friction heat may have an important influence on the sealing performance. Therefore, in this section, the heat dissipation is used to describe the energy consumption of gas face seals with different surface grooves under high-pressure conditions. 

**Figure 12:**
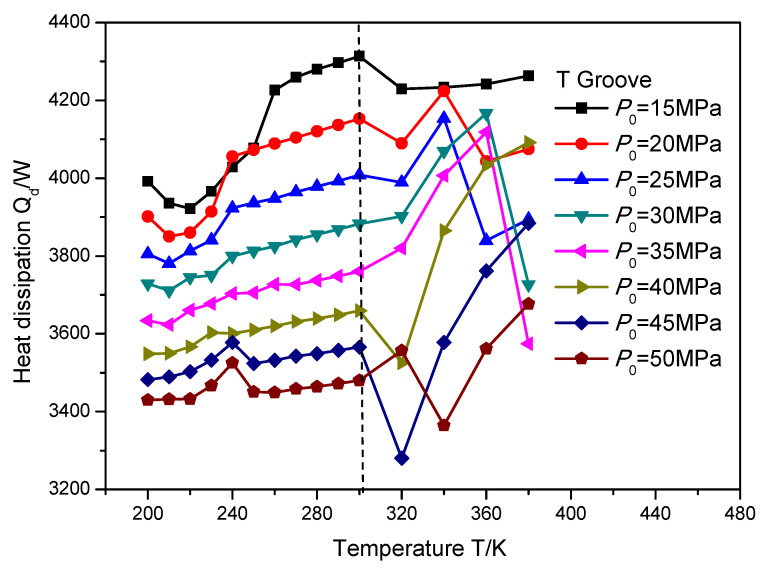
The heat dissipation *Q_d_* of gas face seals with T groove with the increase in temperature and sealing pressure.

**Figure 13:**
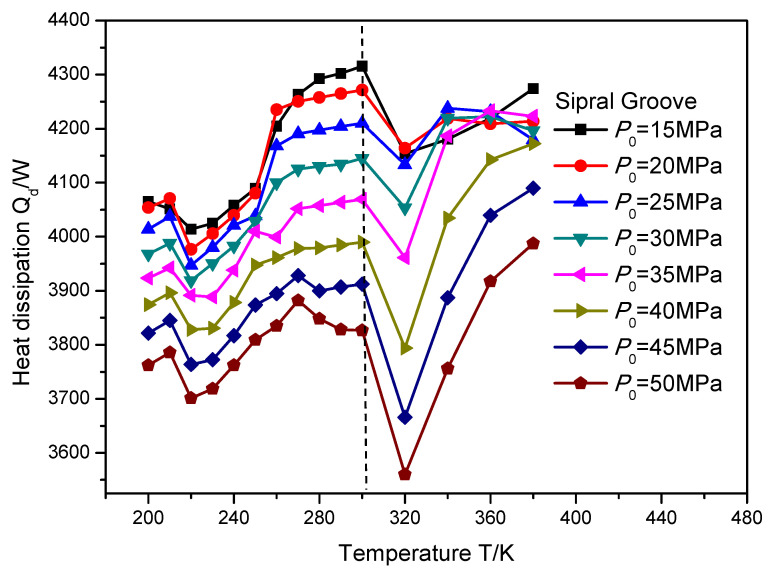
The heat dissipation *Q_d_* of gas face seals with spiral groove with the increase in temperature and sealing pressure.

**Figure 14:**
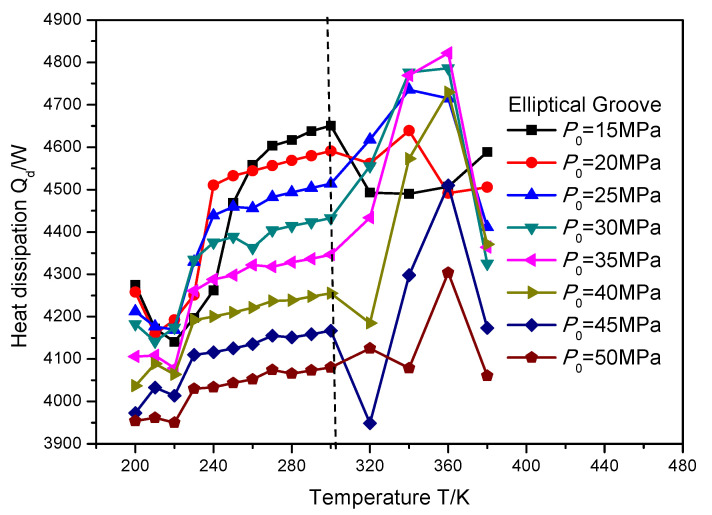
The heat dissipation *Q_d_* of gas face seals with elliptical groove with the increase in temperature and sealing pressure.

In order to investigate the change rule of heat dissipation influenced by the supercritical effect, the temperature and sealing pressure range is set as 200 to 380 K and 15 to 50 MPa, respectively. Figure 12, Figure 13 and Figure 14 illustrate the heat dissipation of the gas face seals with the T groove, spiral groove and elliptical groove with an increase in the temperature and sealing pressure, respectively. It can be seen that when the temperature is less than the supercritical point, the heat dissipation for these three kinds of grooves is consistently changed with the increase in temperature. As a whole, the detailed value of heat dissipation is regularly increased.

When the temperature exceeds the supercritical point, the heat dissipation change of the gas face seals with the spiral groove is also consistent with the growth in temperature, as shown in Figure 13. Specifically, an obvious decrease in heat dissipation will be found first, and then this value will grow consistently. Moreover, the change rate of the heat dissipation becomes larger, from 3.6% to 8.1%, with the increase in the sealing pressure from 15 to 50 MPa, when the temperature grows from 300 to 320 K, while for the T groove and elliptical groove, as shown in Figure 12 and Figure 14, the change rule of the heat dissipation is more complex. This complex change is reflected not only in the monotonicity, but also in the temperature position at which the reflection point occurs. Based on the pervious investigation, this phenomenon may be attributed to the pumping effect and cavitation effect of the different surface grooves. 

## 6. Result Discussion of Sealing Performance

**Figure 15:**
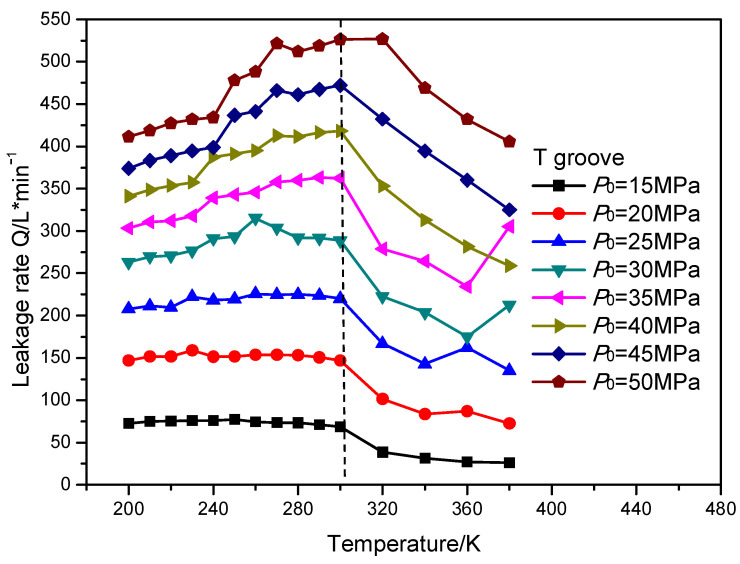
The change rule of leakage rate for gas face seals with T groove with the increase in temperature and sealing pressure.

**Figure 16:**
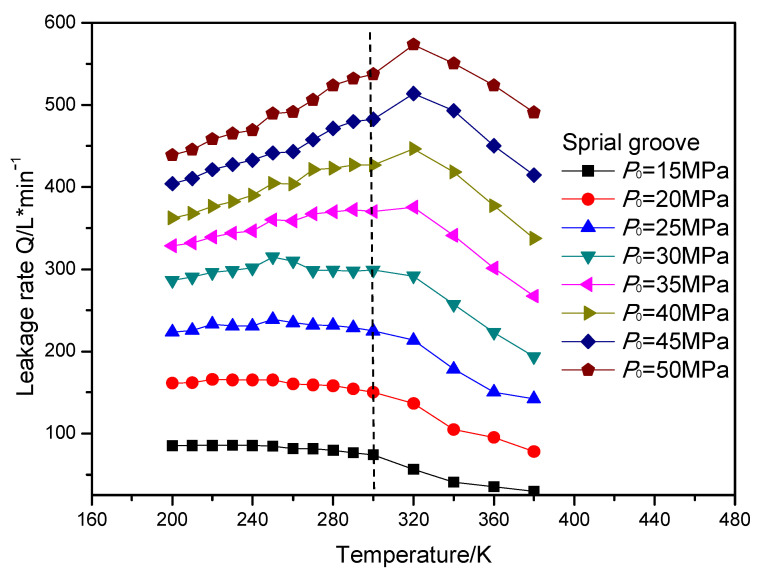
The change rule of leakage rate for gas face seals with spiral groove with the increase in temperature and sealing pressure.

**Figure 17:**
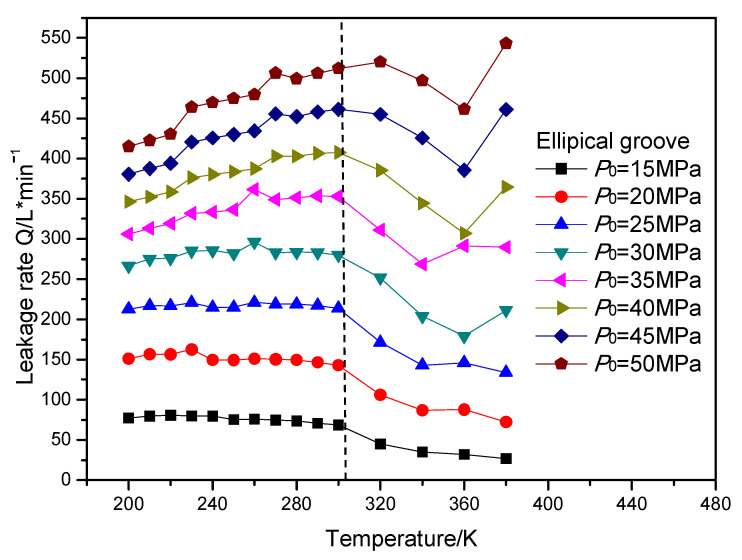
The change rule of leakage rate for gas face seals with elliptical groove with the increase in temperature and sealing pressure.

In this section, the leakage rate and open force of the gas face seals with different surface grooves were calculated. The open force of the gas face seals with diffenent surface grooves remained almost stable with the increasing temperature and sealing pressure. The leakage rate of the gas face seals with the T groove, spiral groove and elliptical groove with the increasing temperature and sealing pressure is shown in Figure 15, Figure 16 and Figure 17, respectively. It can be seen that when the temperature is less than the supercritical point, the leakage rate presents a slowly increasing trend with the growth in temperature. When the temperature is fixed, the leakage rate also increases with the growth in the sealing pressure. Meanwhile, when the temperature is larger than the supercritical point, the leakage rate of the gas face seals with the spiral groove shows an obvious reduction with the growth in the sealing pressure for all pressure cases. However, for the T groove and elliptical groove, the leakge rate may display an obvious increase with the growth in temperature for some pressure cases. The reason for this phenomenon might be that the T groove and elliptical groove are two-direction grooves, which may make the change rule more complex.

## 7. Conclusions

As the mist important structure in the Brayton cycle system, the stability and reliability of the gas face seals have a significant influence on the efficiency of turbines and compressors. In this study, a numerical model is proposed for investigating the flow behavior and energy consumption of face seals under high-temperature conditions. Based on the proposed model, the flow velocity, temperature distribution, heat dissipation and sealing performance are calculated with the increasing temperature and sealing pressure. 

The obtained results show that the supercritical effect of the gas film has a more obvious influence on the flow velocity *u*_θ_ than *u*_r_. Moreover, for three kinds of surface grooves, it was found that the velocity *u*_θ_ and *u*_r_ will be impeded by the groove edge, the velocity *u*_θ_ along the circumference direction remains almost stable and the velocity *u*_r_ shows an obvious reverse flow with the growth in sealing pressure; however, this effect was stronger for the elliptical groove than that for the T groove and spiral groove.

In addition, it was also found that the temperature, heat dissipation and leakage rate of gas face seals presents a dramatic change when the working condition exceeds the supercritical point, which indicates that the thermal properties and sealing performance were unstable near the supercritical point. When considering the design of a sCO_2_ face seal, the working range of the temperature and pressure should avoid the supercritical transition point as far as possible.

The proposed model not only provides insight into the groove design of sCO_2_ face seals, but also provides a theoretical basis for seal designs using other supercritical fluids, like water, hydrogen and helium. Moreover, it could be used in many engineering applications for the reliability analysis of sealing performance due to taking the surface waviness, distortion and temperature-pressure cycles into consideration.

## Figures and Tables

**Table 1:** Parameters of ring materials.

Characteristics	Graphite	Stainless Steel
Materials density/kg·m^−3^	1800	7930
Young’s modulus/G·Pa	25	200
Poisson’s coefficient	0.2	0.3
Specific heat capacity/J·kg^−1^·K^−1^	710	500
Thermal conductivity/W·m^−1^·K^−1^	15	16.2
Linear thermal expansion coefficient/10^−6^ K	4	17.3

**Table 2:** Parameters of working condition.

Item	Symbol	Dimensions and Data
Convection heat transfer coefficient of the stationary and rotating ring at ambient boundaries/W·m^−2^·K^−1^	*k*_gs1_, *k*_gs2_	8.0
Thermal conductivity of gas/W·m^−1^·K^−1^	*k*_c-gas_	0.024
Degrees of freedom of motion of gas molecules	*i*_d_	3
Seal temperature/K	*T*_o_	200~380
Ambient pressure/MPa	*p*_a_	0.1
Sealing pressure/MPa	*p*_o_	15.0~50.0
Basic film thickness/μm	*h*_0_	3.0
Rotational speed/m·s^−1^	*V*	15,000

**Table 3:** Parameters of compared model.

Item	Data	Items	Data
Film thickness	4 um	Outer radius	114 mm
Rotational speed	10,000 r/min	Inner radius	86.2 mm
Sealing pressure	2 MPa	Root radius	102 mm
Ambient pressure	0.1 MPa	Spiral angle	16°
Inlet temperature	356 K	Groove number	16
Initial temperature	293.15 K	Groove depth	8 um

## Data Availability

All data is contained with the article.
